# Production and Characterization of Antioxidative Hydrolysates and Peptides from Corn Gluten Meal Using Papain, Ficin, and Bromelain

**DOI:** 10.3390/molecules25184091

**Published:** 2020-09-07

**Authors:** Ruijia Hu, Gengjun Chen, Yonghui Li

**Affiliations:** Department of Grain Science and Industry, Kansas State University, Manhattan, KS 66506, USA; ruijia@ksu.edu (R.H.); gengjunc@ksu.edu (G.C.)

**Keywords:** corn gluten meal, protein hydrolysates, papain, ficin, bromelain, antioxidants, lipid oxidation

## Abstract

There has been a growing interest in developing natural antioxidants with high efficiency and low cost. Bioactive protein hydrolysates could be a potential source of natural and safer antioxidants. The objectives of this study were to hydrolyze corn gluten meal using three plant-derived proteases, namely papain, ficin, and bromelain, to produce antioxidative hydrolysates and peptides and to characterize the antioxidant performances using both chemical assays and a ground meat model. The optimum hydrolysis time for papain was 3 h, and for ficin and bromelain was 4 h. The hydrolysates were further separated by sequential ultrafiltration to 5 hydrolysate fractions named F1 to F5 from low molecular weight (MW) (<1 kDa) to high MW range (>10 kDa), which were further characterized for TPC, free radical scavenging capacity against DPPH and ABTS, and metal chelating activity. The fraction F4 produced by papain (CH-P4), F1 produced by ficin (CH-F1), and F3 produced by bromelain (CH-B3) showed the strongest antioxidant activity and yield, respectively. These three fractions were incorporated into ground pork to determine their inhibition effects on lipid oxidation during a 16-day storage period. The inhibition effect was enhanced with the addition of higher amount of hydrolysate (e.g., 1000 vs. 500 mg/kg). The CH-P4 reduced lipid oxidation in ground meat by as much as 30.45%, and CH-B3 reduced oxidation by 27.2% at the same level, but the inhibition was only 13.83% with 1000 mg/kg of CH-F1. The study demonstrated that CGM protein hydrolysates and peptides could be used as naturally derived antioxidant in retarding lipid oxidation and improving product storage stability.

## 1. Introduction

Corn is one of the most important food and industrial crops in the world. It typically contains 10–15% protein. The major seed proteins in corn are zein (68%) and glutelin (28%) [1]. Protein quality in corn is poor due to relatively low content of certain essential amino acids, such as Lys and Trp [1,2]. Corn gluten meal (CGM) is a protein-rich coproduct generated during corn wet-milling, containing 60–70% proteins [3]. It is traditionally used as feed materials or otherwise underutilized due to poor protein quality and lack of desired functional properties. Modification of proteins in CGM will broaden its applications and add additional values.

In recent years, studies have showed that various proteins from low value sources such as poultry industry residues [4], fish byproducts [5], and algae waste [6] could be used to produce bioactive and functional protein hydrolysates and peptides. Several approaches are available to produce protein hydrolysates, such as enzymatic or chemical hydrolysis, microbial fermentation. Chemical hydrolysis is conducted using chemical reagents (acid or alkaline), but the protein will be randomly hydrolyzed, leading to variation in peptide compositions from different batches [7,8]. Microbial fermentation is a promising method for the production of bioactive peptides, but it is less efficient [9]. Enzymatic hydrolysis is a predominate method to produce protein hydrolysates with high efficiency and low safety concerns [10]. Some studies have demonstrated that corn protein peptides or domains possess reasonable bioactive functions, such as antioxidant, antihypertension, and anti-obesity activities [11,12,13]. Microbial proteases, such as Alcalase, Protamex, and Flavorzyme were used for CGM hydrolysis [10,14,15]. Plant-sourced enzymes such as papain were used for the hydrolysis of Atlantic salmon skin collagen [16] and sea urchin [17], which lead to antioxidant peptides. However, little information has yet been available on utilizing plant proteases to produce bioactive hydrolysates and peptides from CGM.

Therefore, the primary objective of this study was to produce antioxidative hydrolysates from CGM using three plant enzymes (papain, ficin, and bromelain). The second objective was to evaluate the antioxidant activities of ultrafiltrated fractions with different molecular weight (MW) ranges and to identified peptide sequences of the fractions with promising antioxidant properties. Finally, selected peptide fraction from each hydrolysate was applied into ground meat to evaluate its performance in inhibiting lipid oxidation.

## 2. Results and Discussion

### 2.1. Effect of Hydrolysis Time on Antioxidant Production with Papain, Ficin, and Bromelain

To determine the optimum hydrolysis time for each enzyme (i.e., papain, ficin and bromelain), CGM was hydrolyzed at different reaction times from 0.5 to 5 h. Antioxidant yield, DH, TPC, as well as DPPH radical scavenging activity of the hydrolysates were measured. The optimum hydrolysis time was determined as the time which leads to the most promising antioxidant peptides with regards to antioxidant activities and yield.

**Figure 1:**
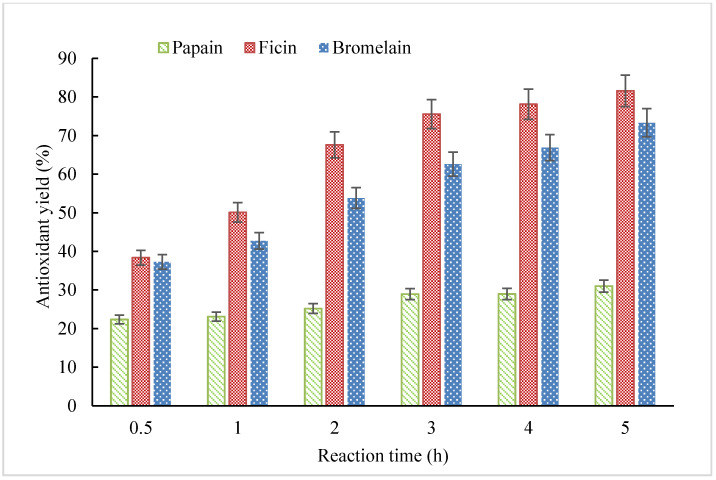
Antioxidant yield of corn gluten meal (CGM) hydrolysates with different reaction times prepared by papain, ficin, and bromelain.

Hydrolysis of CGM was performed only up to 5 h in this study considering economic efficiency. Overall, antioxidant yield increased as the hydrolysis time prolonged for CGM hydrolysates prepared by papain (CH-P), ficin (CH-F), and bromelain (CH-B) (Figure 1). Comparing the antioxidant yield among different types of CGM hydrolysates prepared under the same hydrolysis time, ficin was the most efficient enzyme leading to the highest antioxidant yield than the other two enzymes, while papain was the least efficient. For CH-F and CH-B, antioxidant yield increased rapidly from 0.5 to 3 h, which was approximately 43% and 41%, respectively. However, the yield from 3 to 5 h only increased by 6% and 11% for CH-F and CH-B, which indicated that the hydrolysis reached a stable stage and extending hydrolysis time was not necessary.

**Figure 2:**
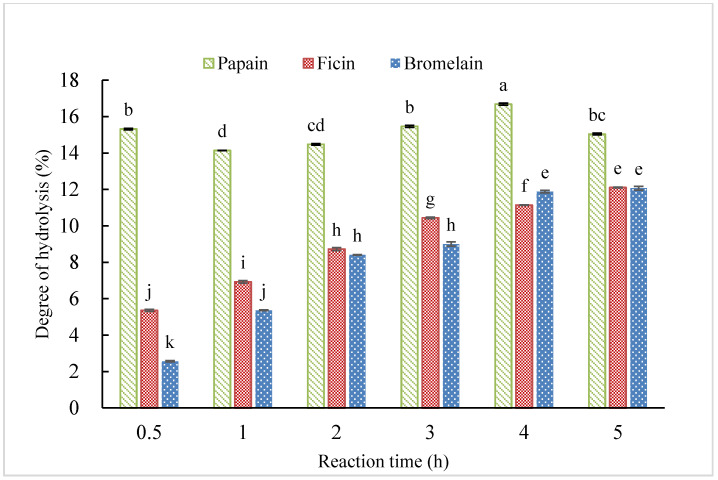
Degree of hydrolysis of CGM hydrolysates under different reaction times prepared by papain, ficin, and bromelain. (Different letters indicate significant differences at *p* < 0.05 for each enzyme).

Figure 2 shows the degree of hydrolysis of each hydrolysate. The DH is defined as the percentage of cleaved peptides bonds after hydrolysis, and it is a critical factor which contributes to the composition and functional properties of the peptides [5,18]. Comparing DH between different types of CGM hydrolysates prepared under the same hydrolysis time, DH of CH-P was significantly higher than that of CH-F and CH-B. This indicated that papain was more efficient to cleave corn peptide bonds. Comparing the DH among the CGM hydrolysates with different hydrolysis times, DH of CH-F and CH-B gently increased with time prolonged and reached the highest DH value of 12.1% at 5 h. The difference of efficiency among the three enzymes may be caused by their different specificities. Papain, bromelain and ficin are the most common endopeptidases from plant sources [19,20]. Papain is a monothiol cysteine endoprotease [21]. Bromelain are proteolytic enzymes and prefer to break down proteins to poly- and oligopeptides [21]. Wharton [22] reported the specificities of bromelain in breaking peptide bonds at nonterminal amino acid. Selamassakul et al. [23] also confirmed that bromelain exhibits specificity in both hydrophobic and nonpolar amino acid residues. Ficin is also a proteolytic enzyme has cleavage specify in tyrosine and phenylalanine bonds [21,24].

**Figure 3:**
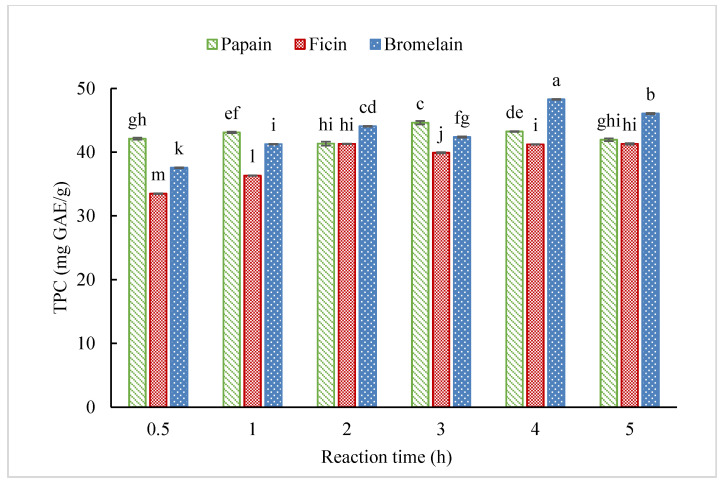
Total phenolic content (TPC) of CGM hydrolysates at 1 mg/mL under different reaction times prepared by papain, ficin, and bromelain. (Different letters indicate significant differences at *p* < 0.05 for each enzyme).

Total phenolic content and DPPH radical scavenging activity were used to evaluate the antioxidant potential of each hydrolysate. TPC of the hydrolysates under different reaction times is shown in Figure 3. Generally, increasing hydrolysis time slightly increased the total phenolic content, due to the release of peptides containing phenolic amino acid residues. Overall, CH-F had relatively lower TPC than the hydrolysates from the other two enzymes with the same hydrolysis time. The CH-B showed the highest TPC at 4 h hydrolysis with value of 48.29 mg GAE/g. The 3 h hydrolysis with papain resulted in significantly higher TPC for CH-P of 44.62 mg GAE/g.

**Figure 4:**
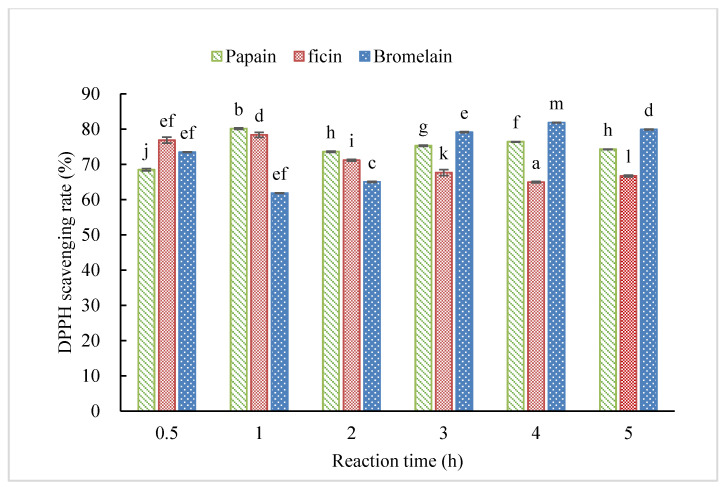
DPPH radical scavenging activity of hydrolysates at 5 mg/mL under different reaction times prepared by papain, ficin, and bromelain. (Different letters indicate significant differences at *p* < 0.05 for each enzyme).

DPPH radical scavenging activity is shown in Figure 4. All hydrolysates exhibited high DPPH scavenging activity with inhibitory rate over 60% even with only 0.5 h hydrolysis, and DPPH activity was not benefited from prolonged hydrolysis. The best DPPH antioxidant capacity was observed for CH-B under 4 h hydrolysis with DPPH inhibition as high as 81.6%, followed by CH-P with 1 h reaction (80.1%). Enzymatic hydrolysis was reported to benefit DPPH scavenging activity of other food proteins, such as milk protein [24], wheat germ protein [25], and rice protein [26]. Hidalgo et al. [27] found that DPPH scavenging of bovine sodium caseinate hydrolysates, which obtained by bacterial proteases produced from *Bacillus* sp. P7, tended to increase with hydrolysis time, while this study showed no clear correlation between hydrolysis time and DPPH scavenging activity for all the hydrolysates from the three enzymes. However, it is difficult to directly compare the antioxidant activities of hydrolysates prepared from different protein sources and by different enzymes due to their different specificity in hydrolysis and different hydrolysate compositions [28].

Considering hydrolysis efficiency, antioxidant activities as well as cost, optimum hydrolysis time for papain was 3 h, and for ficin and bromelain was 4 h. CGM hydrolysates were hydrolyzed under optimum hydrolysis time with each enzyme and further analyzed.

### 2.2. Antioxidant Properties of Ultrafiltrated Hydrolysate Fractions

**Figure 5:**
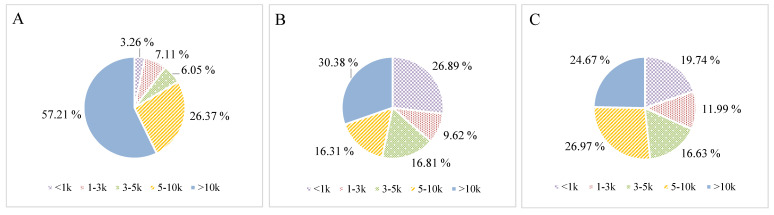
Antioxidant yield of peptide fractions ultrafiltrated from CGM hydrolysates prepared by: (**A**) Papain with 3 h reaction; (**B**) ficin with 4 h reaction; and (**C**) bromelain with 4 h reaction.

Antioxidant activities of protein hydrolysates and peptides were related to their MW [29,30,31]. Prepared CGM hydrolysates were separated into five fractions named F1 to F5 from the lowest MW (below 1 kDa) to the highest MW (above 10 kDa) and evaluated for their antioxidant properties. Figure 5 shows weight distribution of each fraction. For CH-P, F5 (>10 kDa) accounted for most of the hydrolysates (57.21%), and the second largest fraction was F4 (5–10 kDa, 26.37%), followed by F2 (1–3 kDa), F3 (3–5 kDa) and F1 (<1 kDa). The largest fraction of CH-F was also F5 (30.38%), followed by F1, F3, F4, and F2. The largest fraction in CH-B was F4 (26.97%), and the second largest fraction was F5 (24.67%), followed by F1, F3, and F2.

**Figure 6:**
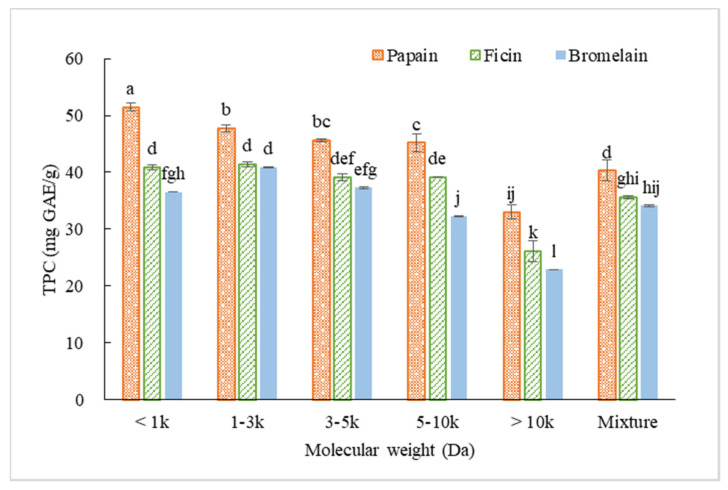
Total phenolic content (TPC) of different peptide fractions (1 mg/mL) ultrafiltrated from CGM hydrolysates prepared by papain (3 h), ficin (4 h), and bromelain (4 h). (Different letters indicate significant differences at *p* < 0.05 for each enzyme).

Total phenolic content of each fraction as well as the crude hydrolysate mixture were measured (Figure 6). Peptide fractions with higher MW exhibited lower TPC. The F1 of CH-P had significantly higher TPC of 51.49 mg GAE/g than the other CH-P fractions. The F2 of both CH-F (41.39 mg GAE/g) and CH-B (40.87 mg GAE/g) possessed higher TPC than their other fractions. For all three types of hydrolysate, F5 exhibited the lowest TPC.

**Figure 7:**
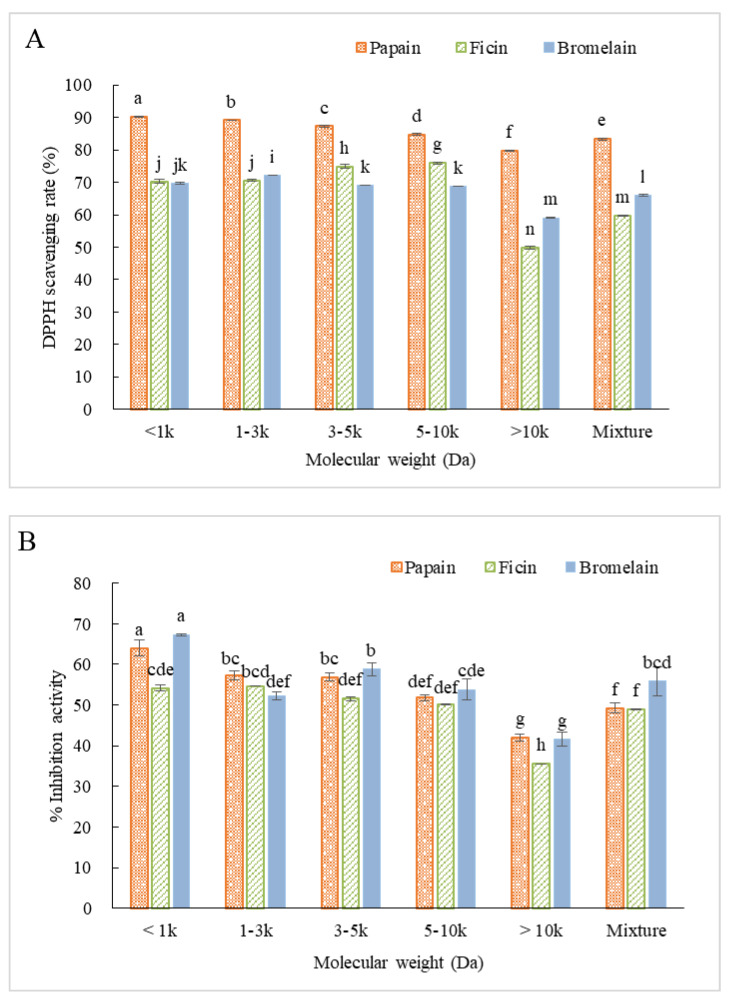

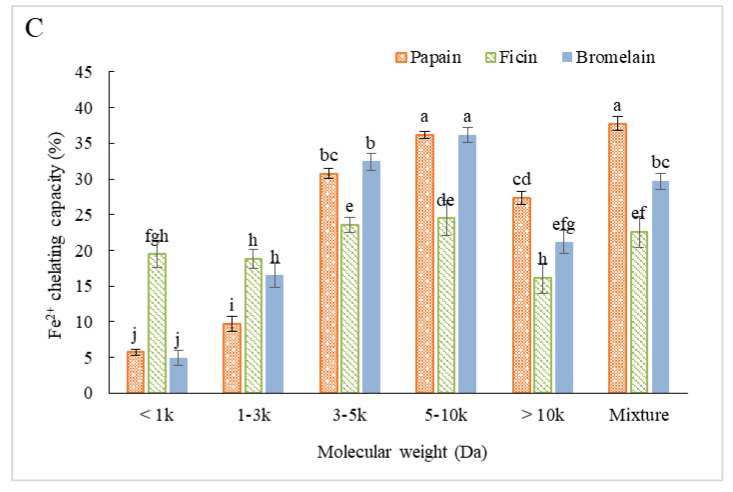
Antioxidant activities of different peptide fractions ultrafiltrated from CGM hydrolysates prepared by papain (3 h), ficin (4 h), and bromelain (4 h). (**A**) DPPH radical scavenging activity at 5 mg/mL; (**B**) ABTS radical scavenging activity at 1 mg/mL; and (**C**) Fe^2+^ chelating activity at 1 mg/mL. (Different letters indicate significant differences at *p* < 0.05 for each enzyme).

Proteins and peptides can perform antioxidant activity through different mechanisms [32]. Hence, the antioxidant capacity of each fraction was evaluated through several assays including DPPH radical scavenging activity, ABTS scanning activity, and metal chelating capacity. DPPH radical scavenging activity of peptide fractions is illustrated in Figure 7A. For CH-P, peptides with lower MW exhibited significantly higher DPPH scavenging activity, and the highest value was observed for F1 (90.1%). Medium sized peptides of CH-F showed better DPPH scavenging capacity, and the highest value was observed for F4 (76.0%), followed by F3 (74.9%). The F2 of CH-B existed the highest scavenging capacity (72.1%) among all the CH-B fractions, and there was no significant difference between F1, F3, and F4, but the values were higher than the crude hydrolysate mixture. Overall, CH-P showed relatively higher scavenging capacity against DPPH than CH-F and CH-B. It was also reported in several other studies that antioxidant activities are related to peptide size, and shorter chain peptides generally have higher antioxidant activity than the longer ones [10,33].

ABTS scavenging activity of ultrafiltrated fractions was also measured (Figure 7B). For CH-P, F1 revealed significantly higher inhibition activity (64.0%) than the other fractions. No significant differences were observed between F2 and F3, as well as between F4 and the mixture. The lowest inhibition of CH-P was found in F5 (42.1%). There was no significant difference of ABTS inhibition observed among the fractions of CH-F, except for F5 with the lowest inhibition rate of 35.6%. The F1 of CH-B had the highest inhibition activity of 67.3%, followed by F3 (58.9%) and the mixture (55.8%). The results of ABTS and DPPH scavenging activities were not completely in agreement, which may be due to the distinct solubility of ABTS radicals (water-soluble) and DPPH radicals (oil-soluble), and different stereoselectivity of the radicals [34]. Overall, small-sized peptides were considered possessing better antioxidant activity against DPPH and ABTS radicals. Peptides perform their antioxidant activity by serving as a proton donor to free radicals [12]. Previous study showed that peptides with lower molecular weight had higher chance of accessibility in order to be adsorbed to the oxidative agents [35,36].

According to Figure 7C, higher metal chelation capacity was observed for medium sized MW fractions for all the three types of hydrolysates. For CH-P, the F4 (36.2%) and hydrolysate mixture (37.8%) exhibited the highest metal chelating capacity. For CH-B, both F3 (24.6%) and F4 (24.5%) showed obviously higher chelating activity with no significant differences. The highest chelating activity of CH-B was observed for F4 with chelation value of 36.2%. As previously reported, the MW of peptides was found to be related to their antioxidant performances [37,38,39]. Zhou et al. [36] reported that the chelation capacity of CGM hydrolysates was highly attributed to smaller MW fractions (500–2500 Da), as well as some bioactive amino acid residues such as Lys, His, Tyr, and Met. Peptides with relatively lower and medium MW were more active as metal ion binder and chelators. Besides, the presence of some amino acid residues could generate extra electrons which improve electrostatic and ionic interaction between themselves and metal ions, such as Asp and Glu, which contributed to strong metal chelation activity especially when they are at the terminal of peptide chain [34,40].

### 2.3. Identification of Peptide Sequences

The F4 from CH-P (CH-P4), F1 from CH-F (CH-F1), and F3 from CH-B (CH-B3) with promising antioxidant activities were identified through RP-HPLC and MALDI-TOF/TOF MS for peptide compositions (Tables S1–S3, Supplementary Document). Numerous peptide sequences were observed for each peptide fraction due to the complex protein composition in CGM, and there were high levels of Glu, Pro, Ala, Leu, Phe, and Tyr in all peptides which agreed with the studies of Li et al. [41] and Hu et al. [42]. Antioxidant properties of protein hydrolysates were related to their composition and structure. Zhuang et al. [31] reported that Leu-Pro-Phe, Leu-Leu-Pro-Phe, and Phe-Leu-Pro-Phe from CGM had high radical-scavenging capacities for ABTS, hydroxyl, DPPH, and superoxide radicals. Besides, corn peptide Tyr-Phe-Cys-Leu-Thr also exhibited excellent antioxidant activities [43]. One possible explanation was the present of specific amino acid residues. The aromatic residues, such as Tyr and Phe, could donate protons to electro-deficient radicals and were usually observed in antioxidant peptides [2,43]. The position of specific amino acids was also critical. For example, Cys residues play an important role as free-radical scavengers when it was in the center of peptide because the thiol group could interact with radicals directly [44].

### 2.4. Inhibition of Lipid Oxidation in Ground Pork

**Figure 8:**
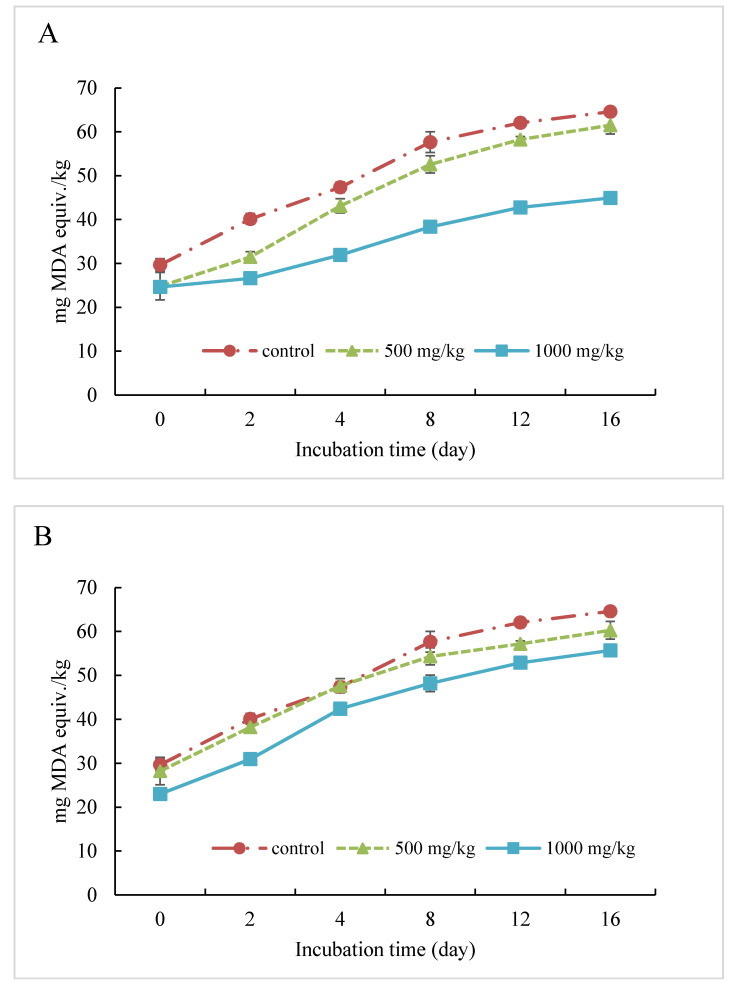

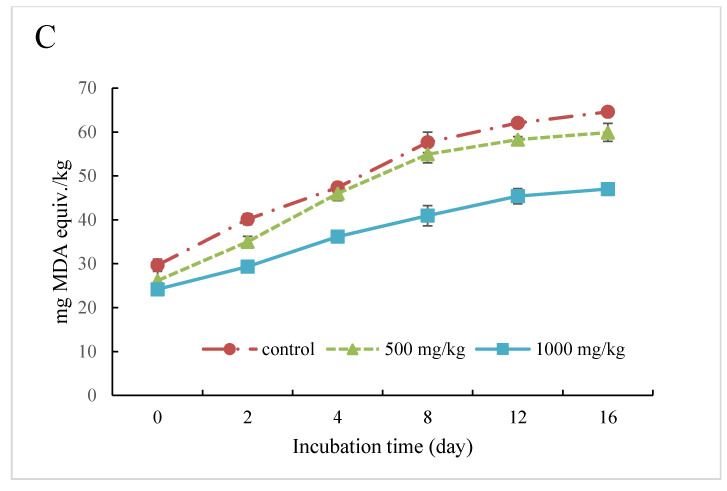
Thiobarbituric acid reactive substance (TBARS) value of selected peptide fractions at 0 (control), 500, and 1000 mg/kg in ground pork. (**A**) 5–10 kDa fraction ultrafiltrated from CGM hydrolysates prepared by papain (CH-P4); (**B**) <1 kDa fraction ultrafiltrated from CGM hydrolysates prepared by ficin (CH-F1); and (**C**) 3–5 kDa fraction ultrafiltrated from CGM hydrolysates prepared by bromelain (CH-B3).

The selected peptide fractions (CH-B3, CH-F1, and CH-P4) were applied in ground pork to further validate their antioxidative performances. The fresh ground pork samples with antioxidant peptides were incubated at 4 °C, and TBARS was measured during 16 days storage. As shown in Figure 8, TBARS values gradually increased from day 0 until the end of storage, and the value for the control sample (no hydrolysates) increased from 29.68 to 64.59 mg MDA equiv./kg. For all the three fractions, meat with addition of 1000 mg/kg peptide antioxidant demonstrated better stability against lipid oxidation than the 500 mg/kg level and the control. The CH-P4 peptide fraction showed the best inhibition against pork lipid oxidation with oxidation reduction as high as 41.9% on day 16 compared with the control, followed by CH-B3 with 34.6% reduction. CH-F1 was the weakest in the protection of lipid oxidation with only 6.5% reduction compared with the control. Zhou et al. [45] studied corn protein hydrolysates prepared by both Neutrase and Alcalase in fresh beef and found that the 1–3 kDa fraction from Neutrase exhibited better protection at both 250 and 500 mg/kg. Researchers showed that some other plant or animal proteins could also be potential source of antioxidative peptides, such as sorghum kafirin [37,46], soy protein [47], fish protein [48], and milk protein [39]. Those bioactive peptides could be used as alternative antioxidant in food systems to prevent lipid oxidation due to the chelating effect of the pro-oxidative metal ions as well as scavenging free radicals [49]. In addition, they could effectively exhibit antioxidant activity in meat system by forming a physical barrier to prevent pro-oxidants approaching the lipid [50].

## 3. Experimental Section

### 3.1. Materials

Corn gluten meal (CGM, 61.3% crude protein) was provided by Grain Processing Corporation (Muscatine, IA, USA). Papain (from papaya latex, crude powder, 1.5–10 U/mg) was purchased from Sigma-Aldrich (St. Louis, MO, USA). Ficin (from figs latex, lyophilized powder, 680 MCU/mg) was purchased from TCI America Co. (Portland, OR, USA). Bromelain (1200 GDU/g, from stem, lyophilized powder) was purchased from Acros Organics (Fairlawn, NJ, USA). All other chemicals, solvents, and reagents used were of analytical grade and purchased from Sigma-Aldrich (St. Louis, MO, USA) or Fisher Scientific (Fairlawn, NJ, USA).

### 3.2. Preparation of Corn Gluten Meal Hydrolysates

CGM was first pretreated to remove water soluble fractions and fats. The CGM was mixed with deionized (DI) water (1:6, *w*/*v*) at room temperature for 1 h, which was then filtrated to remove the water-soluble fraction. The process was repeated twice. The soluble-removed CGM sample was dried in an oven at 45 °C for 48 h. Fat was removed by mixing the dried CGM with hexane (1:6, *w*/*v*) for 0.5 h, which was then filtrated to remove the solvent containing fat. The process was repeated three times. The defatted CGM was placed in a fume hood for at least 24 h to completely volatilize the hexane. CGM suspension (4%, *w*/*v*, protein base) was prepared by dispersing the pretreated CGM in 250 mL DI water. The CGM suspension was heated in a 95 °C water bath for 10 min to denature the proteins and enhance hydrolysis efficiency. The pH of the suspension was then adjusted to the desired level when it was cooled down to room temperature. Enzymatic hydrolysis was conducted in a water bath shaker with optimum temperature for each enzyme. The enzyme-to-substrate ratio, pH, temperature, and reaction time used for the three enzymes are listed in Table 1. At the end of the reaction, the mixture was heated again to 95 °C to denature the protease, cooled down, and centrifuged to collect the supernatant, which was then lyophilized and stored at −20 °C until further analysis.

### 3.3. Determination of Antioxidant Yield

The yield of antioxidant was calculated as the ratio of soluble fraction after hydrolysis using the equation as follow: Antioxidant yield = (W_2_/W_1_) × 100%, where W_1_ was the amount of protein in CGM used for hydrolysis, and W_2_ was the amount of lyophilized hydrolysate supernatant.

### 3.4. Determination of Degree of Hydrolysis (DH)

The DH of CGM hydrolysates was determined by o-phthaldialdehyde (OPA) assay according to a previously established protocol [51]. Serine (0.9515 mM) was used as standard. Hydrolysate samples were measured at a concentration of 1.2 mg/mL.

### 3.5. Fractionation of CGM Hydrolysate by Ultrafiltration

CGM hydrolysates were fractionated by an Amicon^®^ Stirred Cell device (EMD Millipore Corporation, Billerica, MA, USA) under pressure of nitrogen (60 psi) with continually stirring on a magnetic stirrer (60 rpm). Separation of hydrolysates was conducted based on molecular weight using ultrafiltration membranes (EMD Millipore Corporation, Billerica, MA, USA) with different cut-off sizes (1, 3, 5, and 10 kDa). The eluent fractions were lyophilized and stored at −20 °C until further analysis.

### 3.6. Determination of Total Phenolic Content (TPC)

TPC of CGM hydrolysates at 1 mg/mL was evaluated based on Folin–Ciocalteu method according to Thamnarathip et al. [52]. Gallic acid (0–0.06 mg/mL) was used as a standard. Total phenolic content of hydrolysates was expressed as mg gallic acid equivalent per gram of sample (mg GAE/g).

### 3.7. Determination of Antioxidant Activity

#### 3.7.1. Determination of DPPH Radical Scavenging Activity

The scavenging activity of CGM hydrolysates on 1.1-diphenyl-2-picrylhydrazyl (DPPH) free radical was measured according to the modified method of Li et al. [12]. Briefly, 5 mL of DPPH solution (0.2 mM) in 95% ethanol was added into 5 mL hydrolysate solution (5 mg/mL). The mixture was vortexed for 15 sec and rested in dark for 30 min, and the absorbance was measured at 517 nm. DI water instead of sample solution with the same treatment was used as blank. The DPPH radical scavenging activity was expressed as follows:

DPPH scavenging rate (%) = [(A_blank_ − A_sample_)/A_blank_] × 100 (1)

#### 3.7.2. Determination of ABTS Radical Scavenging Activity

ABTS radical scavenging activity of hydrolysate solution at 1 mg/mL was determined following a previous method reported [53]. DI water instead of sample was used as the blank. The ABTS radical scavenging activity was calculated using the equation as follows:

ABTS scavenging rate (%) = [(A_blank_ − A_sample_)/A_blank_] × 100 (2)

#### 3.7.3. Determination of Ferrous ion (Fe^2+^) Chelating Activity

The Fe^2+^ chelating activity was assessed according to a previously reported protocol with slight modifications [32]. Briefly, 25 μL of hydrolysate (1 mg/mL), 150 μL of DI water, and 25 μL of FeCl_2_ solution (0.2 mM) were loaded into microcell plate. After incubating at room temperature for 30 s, 50 μL of ferrozine solution (1 mM) was then added into the mixture, and the absorbance was read at 562 nm. DI water was used as blank. The chelating ability was calculated as follows:

Fe^2+^ chelating ability (%) = [(A_blank_ − A_sample_)/A_blank_] × 100 (3)

### 3.8. Identification of Peptide Sequences of Selected Antioxidant Peptide

Peptide fractions with promising antioxidant properties as well as desirable yield were selected for peptide sequence analysis using an Ultraflex ΙΙΙ Matrix-assisted Laser Desorption Ionization-Time of Flight/Time of Flight Mass Spectrometry (MALDI-TPF/TOF MS) (Bruker Daltonik GmbH, Bremen, Germany) [37].

### 3.9. Antioxidant Activity of Selected Hydrolysates in Ground Pork

The inhibition effects of selected CGM hydrolysates on lipid oxidation in ground pork was determined based on thiobarbituric acid reactive substance (TBARS) assay following a previously reported protocol of Zhang, Li, and Zhou [54] with some modifications. Meat sample was prepared by mixing 50 g ground pork with 5 mL hydrolysate solutions (5 and 10 mg/mL) and three drops of 0.2% sodium azide. Prepared meat samples were stored at 4 °C during analysis. To extract the oxidation products, 5 g of the prepared meat was homogenized with 50 mL DI water, 10 mL reducing agent (0.01% propyl gallate, 0.02% EDTA), and 0.1 mL sodium dodecyl sulfate (SDS, 10%,) for 2 min. The homogenate (1 mL) was transferred into a 15 mL tube and mixed with 4.0 mL TBA solution (0.4% TBA, 0.5% SDS, and 9.3% acetic acid), and then reacted in a 95 °C water bath for 1 h. The mixture was cooled down in cold water for 10 min, and 5 mL of pyridine/butanol (1:15, *v*/*v*) was added. Following centrifugation at 3500× *g* for 15 min, the top layer was collected, and the absorbance was measured at 532 nm. The 1, 1,3,3-tetramethoxypropane (TMP) solutions (0 to 10 μM) were used as standard, and result was expressed as mg malonaldehyde (MDA) equivalent per kilogram of meat (mg MDA equiv./kg).

### 3.10. Statistic Analysis

All the experiments were conducted in triplicate. Results were analyzed with SAS 9.3 software (SAS Institute, Cary, NC, USA). One-way analysis of variance (ANOVA) was performed, and Tukey’s post-hoc test was used to determine significant differences between the means, which are considered significant at *p* < 0.05. The presented data are the mean values of three replicates, and the error bars indicate the standard deviation (*n* = 3).

## 4. Conclusions

The hydrolysates prepared from CGM using papain, ficin and bromelain showed different yield, degree of hydrolysis, and antioxidant properties, and this indicated that the antioxidant activity of CGM hydrolysate is highly dependent on enzyme types, as well as hydrolysis conditions. The antioxidant activities of CGM hydrolysates were also MW dependent. Lower size peptide fractions exhibited better antioxidant activities. Considering both yield and antioxidant activities, both bromelain and ficin are recommended for producing CGM hydrolysates with high antioxidant activities. Application of antioxidant peptides in ground pork at 1000 mg/kg efficiently inhibited lipid oxidation. This study demonstrated that CGM could be a potential source to produce antioxidant hydrolysates and peptides, and ficin and bromelain could serve as efficient enzymes to hydrolyze CGM proteins and improve its antioxidant functionality. The antioxidative hydrolysates and peptides can be used as an alternative antioxidant in various foods, pet food, and animal feed to prevent lipid oxidation and improve product storage stability.

## Figures and Tables

**Table 1:** Parameters for enzymatic hydrolysis of corn gluten meal.

Enzyme Type	Enzyme Amount, mg/g of Protein	pH	Temperature, °C	Time, h
Papain	40	6.5	50	0.5–5
Ficin	225.5	6.0	50	0.5–5
Bromelain	150	5.0	50	0.5–5

## Supplementary Materials

The following are available online, Table S1: Peptide sequences of 3–5 kDa peptide fraction ultrafiltrated from bromelain-hydrolyzed CGM with 4 h hydrolysis; Table S2: Peptide sequences of <1 kDa peptide fraction ultrafiltrated from ficin-hydrolyzed CGM with 4 h hydrolysis; Table S3. Peptide sequences of 5–10 kDa peptide fraction ultrafiltrated from papain-hydrolyzed CGM wth 3 h hydrolysis.
