# Supplementary material for: Production and Characterization of Antioxidative Hydrolysates and Peptides from Corn Gluten Meal Using Papain, Ficin, and Bromelain
Source: Molecules. 2020 Sep 7;25(18):4091. doi: 10.3390/molecules25184091 (PMC7571122; doi:10.3390/molecules25184091)
Supplement: Supplementary file 1 [file molecules-25-04091-s001.pdf]

Table S1. Peptide sequences of 3-5 kDa peptide fraction ultrafiltrated from bromelain-hydrolyzed CGM with 4 h hydrolysis.

	RP-HPLC peak retention time	4.0 min	5.55 min	6.5 min	8.1 min	12.3 min	13.1 min	14.5 min	15.6 min	19.1 min	23.03 min	24.9 min	41.9 min
Zein-16	Area%	21.1%	48.6%	10.6%	9.6%	80.2%	11.2%	23.3%	6.4%	45.0%	4.1%	8.5%	45.8
	Coverage%	15.8%	16.4%	8.7%	8.2%	14.2%	19.1%	15.3%	12.6%	71.0%	69.9%	43.2%	22.4
	Sequences	VALAL	GC	KVLIVALAL	MKVLI	RH	KVLIVALAL	VALAL	QQCC	MKVLI	KVLIVALAL	IVALAL	ASAASS
		LALAASA	TP	FYLPP	QQ	CS	LALAA	QQF	ATY	SGGCGC	LALAASAASS	LALAASAASS	TS
		FYM	VGSVGS		QP	LQ	GGCGC	GSCGVGSVGS	GVVL	TP	TSGGCGC	TSGGCGC	LPP
		PPPFYL	RH		GELAA	QPQGELA	TP	PFLG	QQCC	PFHLPPPFYM	TP	PFHLPPPFYM	HRY
		CH	CS			AQQLTA	QQCC	IR	QP	PPPFY	PFH	PPPFY	LTA
		IR	GVVL				ATY		GELAA	SPC	YM	SVGS	PCNAAAG
			GELA				GVVL			GSCGVGSVGS	PPPFYLPP	PFLG	
										PFLG	PW	CSPAATP	
										CVEFL	YP	YGSP	
										RH	PP	GL	
										PAATP	LSPC	PG	
										YGSP	GSCGVG	PCPCNA	
										CCH	IR		
										EPLHRY	EPLHRY		
										ATY	ATY		
										GVVL	GVVL		
										SFL	SFL		
										VA	ALM		
										LTA	AA		
										MCGL	VA		
										CPCNAAAGG	LTA		
										VYY			
Zein-19	Area%	26.3%	27.6%	17.8%	21.5%	75.2%	24.3%	56.6%	16.2%	35.4%	5.4%	5.9%	14.2
	Coverage%	23.3%	8.8%	16.2%	26.7%	22.1%	13.1%	30.8%	12.5%	61.2%	76.5%	39.6%	10
	Sequences	LLM	KIFSLLM	KIFSLLM	MATKIFSLLM	RLQQ	KIFSLLM	KIFSLL	KIFSLLM	CVAN	KVLIVALAL	IFSLLM	MATKIFSLL
		LLM	LLALSTC	LLALSTC	LLALSTC	ASNIPLS	LLALSTC	LLALSTCVAN	LLALSTC	ATIFPQCSQA	LALAASAASS	LLALSTC	LLALSTC
		LLPPYL	HIIGGAL	IFPQCS	PIASLLPP	AL	QLVL	ATIFPQC	AIAASNIPL	PIASLLPPY	TSGGCGCQTP	ATIFPQCSQA	PIASLLPP
		PSII		QLVL	SIIASI	SLVQ	LLPFY	AIAASNIPLS	ALAN	SIIACICEN	PFH	PIASLLPPY	
		QQ		PL	IAASNIPL	RAQQL	HIIGGAL	AL	LSP	PALQPYR	YM	PSIIA	
		AIAASNIP		FL	ALAN	NQVALAN		SLVQ		AIAASNIPLS	PPPFYLPPQQ	LVQTI	

		FNQLSTLN<br>AYS<br>QQQQLLP<br>SFL<br>TQQLLP		TQQQ<br>HIIGGAL	LLPFN<br>LAALNPAA<br>LPFVQ	QQILL<br>LAAANR<br>LL<br>QLQQLL		LSPYS<br>QQILL<br>LLPFY<br>TLL<br>QLQQLLP<br>HIIGGAL		PL<br>LVQTI<br>RAQQL<br>NQVALAN<br>LSPYSQQQQF<br>LPFNQLSTLN<br>PAAYLQQQLL<br>PFSQLATAYS<br>QQQQLLPFNQ<br>LAAANRASFL<br>TQQ<br>TLL<br>QLQQLLPFVQ	QPQPWQYP<br>PPQLSPCQQF<br>GSCGVGSVGS<br>PFLGQCV<br>IRQV<br>EPLHRYQATY<br>GVVLQSFLQQ<br>QP<br>ALM<br>AAQVAQQLTA<br>MCGLQLQQPG<br>PCPCNAAAGG<br>VY	RAQQLQQLV<br>AAYLQQQLL<br>PFSQLATA<br>PAAYL<br>QQQILLP<br>PAASY<br>QQQHIIGGAL	
Glutenin	Area%	52.9%	46.6%	5.3%	20.2%	100.0%	11.2%	42.1%	5.9%	29.0%	5.8%	7.1%	12.1%
	Coverage%	39.9%	8.5%	4.0%	15.2%	24.2%	12.1%	20.2%	15.7%	63.2%	68.6%	29.6%	12.6%
	Sequences	VALAL<br>LALAASATST<br>HTSG<br>PILG<br>LRH<br>CSPTA<br>PYCSP<br>LRH<br>HRY<br>GLVL<br>VALAL<br>GLLAA<br>IA<br>MCGL<br>TPCPYAAAG	GVG<br>STPILG<br>QVA<br>GLLAA	VLLVALAK	AL<br>LALAASATST<br>PHP<br>CP<br>GLLAA<br>GLLAA<br>TP	HP<br>PH<br>CQQPH<br>GVG<br>STPILG<br>RH<br>CS<br>QS<br>LR<br>QQQPQSG<br>VA<br>GLLAA<br>LTA	LAL<br>LALAA<br>QPP<br>RP<br>QQCCQQL<br>TPCPYAA	VLLVALA<br>GTCGVG<br>STPILG<br>PTA<br>TPYCSP<br>IFG<br>VA<br>GLLAA	HVPPPV<br>QCC<br>QL<br>SG<br>VA<br>GLL<br>TAQ<br>LTA<br>TPCPYAA	LAASATS<br>HTSGGCG<br>PPVHLPPPV<br>HVPPPVHLPP<br>PPCH<br>RP<br>PHP<br>CPC<br>PHPSP<br>GTCGVG<br>STPILG<br>PTA<br>TPYCSP<br>LR<br>CC<br>IFGLVL<br>SIL<br>LLAA<br>IA<br>LTAMCGL<br>TPCPYAA	LVALAL<br>LALAASATST<br>HTSGGCG<br>CPC<br>PHPSP<br>GTCGVG<br>STPILG<br>CVE<br>FIRH<br>CSPT<br>YA<br>LTAMCGL<br>TPCPYAAAG	AL<br>LALAASATST<br>HTSGGCGC<br>VHLPPPV<br>HVPPPVHLPP<br>PPCHYP<br>SP<br>GTCG<br>VL<br>SIL<br>SG	SATST<br>HVPPPV<br>SG<br>VA<br>GLL<br>IA<br>LTA

Table S2. Peptide sequences of <1 kDa peptide fraction ultrafiltrated from ficin-hydrolyzed CGM with 4 h hydrolysis

	RP-HPLC peak retention time	4.1 min	6.4 min	12.9 min	14.2 min	15.9 min	17.5 min	19.3 min	19.9 min	21.7 min	23.03 min	41.9 min	51.3 min
Zein-16	Area%	23.5%	44.5%	6.0%	9.2%	11.8%	3.9%	19.9%	20.7%	18.0%	13.4%	2.0%	14.0%
	Coverage%	43.7%	18.6%	8.7%	51.9%	23.0%	67.2%	79.8%	65.0%	30.1%	31.7%	13.1%	60.7%
	Sequences	KVLIVALAL	GCQTP	LTA	MKVLI	VLIVALAL	MKVLIVALAL	MKVLIVALAL	KVLIVALAL	KVLIVALAL	AASAASS	SVGS	ALAASAASS
		LALAA	GQCVEF	MCGL	LPPPFYM	AASS	LALAASAASS	LALAAS	LALAASAASS	LALAASAASS	TS	PFLG	TSGGCGC
		GGCGCQTP	RHQCS	PG	PPPFYLPP	TSGGCGC	TSGGCG	TSGGCGCQTP	TSGGCGCQTP	TSG	GCQTP	QCSPAATP	LPPPFYM
		PF	HQIRQ	PCP	GSCGVGSVGS	CQQF	PFYM	PFHLPPPFYM	PFH	QPQPWQYPTQ	LPPQQ	YGSPQC	CGVGSVGS
		PPFYM	GVVLQS		PFLG	GSCGVGSCGS	PPPF	PPPF	FYM	PPQLS	PW		PFLGQCVEFL
		PPFYM	QPQGELA		FL	PAATP	LPPQQ	QLSPCQQF	PPPFYLPP	GSPQCQ	YPTQ		RH
		LPPQQ			PHQCSPA	YG	QPQPWQYPTQ	GSCGVGSCGS	PW	HRY	PP		CSPAATP
		GSCGVGSVG			YGSP		PPQLSPCQQF	PFLGQCVEFL	YPT	PCNAAAG	VGSVGS		YGSP
		GQCVEF			AL		GSCGVGSVGS	RHQCSPAATP	VGSVGS		HRYQ		AL
		LQ			QCCHQIRQV		PFLGQCV	YGSPQCQ	PFLG		GVVL		QQCCH
		QQCC			EPLHRYQATY		GSPQCQAL	QQCCHQTRQV	CVE		PCNAAAG		PLHRY
		QV			GVVLQSFLQ		QQCCHQIR	EPLHRYQATY	GSP				GELAA
		EPLH			ALM		QSFLQ	GVVLQSFLQQ	AL				AQQLTA
		QATY			AA		QPQGELAA	QPQG	QQC				MCG
		GVVLQSF			PCPCNA		CGLQLQQPG	TA	QV				PCNAAAG
		LM					PCPCNAA	MCGLQLQQPG	EPLHRYQATY				VY
		AAQV						PCPCNAAAGG	GVVL				
								VYY	SF				
									LAALM				
									QVA				
									QLTA				
									AA				
Zein-19	Area%	22.8%	52.0%	23.6%	9.6%	2.3%	2.8%	4.0%	3.2%	2.6%	8.6%	11.2%	12.2%
	Coverage%	21.7%	20.0%	28.8%	55.8%	7.9%	61.7%	67.1%	52.9%	69.2%	20.0%	10.8%	29.6%
	Sequences	MATKIFSLLM	KIFSLLM	MATKIFSLLM	KIFSLLM	KIFSLLM	IFSLLM	FSLLM	SLLPPYL	TKIFSLLM	QSPAL	MATKIFSLL	SLLM
		LLALSTC	LLALSTC	LLALSTC	LLALSTCVAN	LLALS	LLALSTCVAN	LLALSTCVAN	PSIIASICEN	LLALSTCV	ALAN	LLALSTC	LLALS
		IFPQCS	AL	TIFPQC	ATIFPQC	AAANRAS	ATIFPQCSQA	ATIFPQCSQA	PALQP	PPYL	LSPYSQQQQF	ATIFPQCSQA	ATIFPQCSQA
		PIASLLPP	SLVQ	PIASLLPP	SIIASI		PIASLLPPYL	PIASLLPPYL	SNIPLS	PSIIASICEN	FNQLSTLN		YRLQQ
		QLVL	QLVL	QLVL	AIAASNIPLS		PSIIASICEN	PSIIASI	PLLFQQSPQL	PALQPYRLQQ	PAAYLLQQ		AIAASNIPL
		PLLN	PL	QQQLL	PLLFQQSPAL		PALQPYRLQQ	AIAASNIPLS	SLVQSLVQTI	AIAASNIPLS	QQQQL		ALAN
		LLPFYQ	QQILLPF	QQQLL	SLVQS		AIAASNIPLS	PL	RAQQLQQLVL	PLLFQQSPAL	TQQQL		LSPYSQQ
		HIIGGAL	LLALSTC	LAALNP	NQLSTLN		PLLFQQSPAL	PAL	PLINQVALAN	SLVQSLVQTI			FNQ

			QLQQLL<br>HIIGGAL	QQQLLP<br>HIIGGAL	PAAYL<br>PFSQLATAYS<br>QQ<br>LLPEN<br>ALNPAAYL<br>QILLPFSQ<br>RASFL<br>TQQQLLPFYQ<br>QFAA<br>TLL<br>QLQQLLPFVQ		SLVQSLV<br>PLINQVALAN<br>LSPYSQQQF<br>LPPQQ<br>PFSQLATAYS<br>QQQQLLPEN<br>NPATLL<br>QLQ<br>PFVQ<br>LALTDPAASY	SLVQSLVQTI<br>RAQQL<br>QF<br>LPFNQLSTLN<br>PAAYLQQQLL<br>PFSQLATAYS<br>QQQQLLPFNQ<br>LAALNPAAYL<br>QQQILLPFSQ<br>AANPATLL<br>QLQQLLPFVQ<br>LALTDPAA	LSPYSQQQQF<br>LPFNQLST<br>QQQILLPFSQ<br>LAAANR<br>LQQLLPFVQ<br>LALTDPAASY<br>QQHIIG	RAQQLQ<br>LVL<br>PLINQVALAN<br>LSPYSQQQQF<br>LPFNQLST<br>QQQQLLPENQ<br>LAAL<br>QILLPFSQ<br>LAAANRA<br>ATLL<br>QLQQLLPFVQ<br>LALTDPAASY<br>QQHIIG			LAALNPA<br>QILLPFSQ<br>ASFL<br>TQQQ
Glutenin	Area%<br>Coverage%<br>Sequences	35.4%<br>27.8%<br>ALAL<br>LALAA<br>QPP<br>QRPQ<br>PQPHP<br>CPC<br>PILGQCVE<br>PYCSPQ<br>QCCQQL<br>QVA<br>GLLAA<br>QPP<br>TPCPYAAA	25.0%<br>5.8%<br>PILGQC<br>QVA<br>GLLAA	28.2%<br>19.3%<br>AASATST<br>HTSGGCGCQP<br>PILGQC<br>IL<br>QVA<br>GLLAA<br>LTAMCGL	5.2%<br>19.3%<br>SATST<br>HTSGGCGCQP<br>PTA<br>TPYCSPQCQS<br>LR<br>QLR<br>QVEP<br>QVEPQHRY	3.0%<br>12.6%<br>VLLVALAL<br>LA<br>TST<br>HTSGG<br>QP<br>PPPVHLPP	3.8%<br>71.3%<br>VLLVALAL<br>LA<br>HLPPV<br>HLPPVHLPP<br>PVHLPPPVHL<br>PPPVHLPPPV<br>VHLPP<br>PPCHYPTQPP<br>PC<br>QPHPSP<br>GTCGVG<br>STPILG<br>CVE<br>FLRHQCSPTA<br>TPYCSPQCQS<br>QQLR<br>QVEPQ<br>QA<br>IFGLVLQSIL<br>QQQPQSG<br>AQIAQQ<br>LTAMCGLQQP<br>**TPCP**	24.8%<br>67.3%<br>VLLVALAL<br>LALAASATST<br>HTSGGCG<br>PPVHLPPPV<br>HVPPPVHLPP<br>PSP<br>CQLQGTCGVG<br>STPILGQCVE<br>FLRHQCSPTA<br>TPYCSPQCQS<br>LRQQCCQQLR<br>QVEPQH<br>QA<br>IFGLVLQSIL<br>QQQPQSGQVA<br>GLLAAQIAQQ<br>LTAMCGLQQP<br>TPCPYAA	26.6%<br>84.8%<br>LLVALAL<br>LALAASATST<br>HTSGGCGCQP<br>PPVHLPPPV<br>HLPPVHLPP<br>PVHLPPPVHL<br>PPPVHLPPPV<br>HVPPVHLPP<br>PPCHYPTQPP<br>PQPHPQPHP<br>CPCQQPHPSP<br>CQLQGTCGVG<br>STPILGQCVE<br>FLR<br>PTA<br>TPYCSPQCQS<br>LRQQCCQQLR<br>QQQPQSGQVA<br>GLLAAQIAQQ<br>LTAM<br>GLQQP<br>TPCPY<br>**AAAG**	7.0%<br>65.0%<br>MRVLLALAL<br>LALAASATST<br>HTSGGCGCQP<br>PPVHLPPPV<br>HLPPVHLPP<br>PVHLPPPVHL<br>PPPVHLPPP<br>ILGQCVE<br>FLR<br>CSPQCQS<br>LRQQCC<br>QHRYQA<br>IFGLVL<br>AQIAQQ<br>LTAMC<br>TPCPYAAGG<br>VP	18.0%<br>27.4%<br>MRVLLALAL<br>LAASATST<br>HTSGG<br>CHYPT<br>RP<br>PHP<br>QGTCGVG<br>STP<br>QHRY<br>PYAAAGG<br>VP	10.0%<br>9.4%<br>VHLPPPV<br>HVPPPVHL<br>VCVE<br>FLR	23.0%<br>28.3%<br>MRVLLV<br>VHLPPPV<br>HVPPPVHLPP<br>PPCH<br>SP<br>GTCGVG<br>ST<br>CVE<br>FLR<br>GLLAA<br>IA<br>LTAMCG

Table S3. Peptide sequences of 5-10 kDa peptide fraction ultrafiltrated from papain-hydrolyzed CGM wth 3 h hydrolysis

Compared peptides	RP-HPLC peak retention time	5.05 min	6.8 min	13.3 min	16.5 min	18.3 min	19.5 min	21.5 min	24.5 min	42.7 min	52.9 min
Zein-16	Area%	11.1%	8.9%	17.6%	20.7%	2.3%	8.3%	37.1%	19.0%	20.5%	34.0%
	Coverage%	47.5%	62.3%	43.2%	89.1%	42.6%	92.9%	98.4%	84.2%	18.6%	84.2%
	Sequences	MKVLIVA<br>PPFYM<br>PPPFYLPPQQ<br>QP<br>PPQLSPCQQF<br>GSCG<br>PFLGQCVEFL<br>RHQC<br>EPLHRYQATY<br>GVVLQSFL<br>QLTA<br>MCGLQLQQPG<br>TQ	VALAL<br>LALA<br>GGCGCQTP<br>PF<br>PPPFYM<br>PPP<br>YLPPQQ<br>PQLSPCQQF<br>GSCGVGSVGS<br>CSPAATP<br>YGSPQCQALQ<br>QQCCHQIRQV<br>EPLHRYQATY<br>AQVAQQLTA<br>MCGLQLQQPG<br>PCN	KVLIVALAL<br>LALAASAA<br>TSGGCGCQ<br>PPPFYLPPQ<br>FLGQCVEFL<br>RYQATY<br>GVV<br>QPQGELAALM<br>QLTA<br>LQQPG<br>PCPCN	MKVLIVALAL<br>LALAASAASS<br>TSGGCGCQTP<br>PF<br>PPPFYLPPQQ<br>QYPTQ<br>PPQLSPCQQF<br>GSCGVGSVGS<br>PFLGQCVEF<br>HQCSPA<br>YGSPQCQALQ<br>QQCC<br>EPLHRYQATY<br>GVVLQSFLQQ<br>QPQGELAALM<br>AAQVAQQLTA<br>MCGLQLQQPG<br>PCPCNAAAGG<br>QV<br>VY	MKVLIVALAL<br>HLPPPFYM<br>PPPFYLPPQQ<br>QPQPW<br>PTQ<br>PPQLSPCQQF<br>GSCG<br>PFLGQCVE<br>TY<br>GVVLQSFL<br>PCPCNA<br>PG	VLIVALAL<br>LALAASAASS<br>TSGGCGCQTP<br>PFHLPPPFYM<br>PPPFYLPPQQ<br>QPQPWQYPTQ<br>PPQLSPCQQF<br>GSCGVG<br>GQCVEFL<br>RHQCSPAATP<br>YGSPQCQALQ<br>QQCCHQIRQV<br>EPLHRYQATY<br>GVVLQSFLQQ<br>QPQGELAALM<br>AAQVAQQLTA<br>MCGLQLQQPG<br>PCPCNAAAG	MKVLIVALAL<br>LAL<br>SAASS<br>TSGGCGCQTP<br>PFHLPPPFYM<br>PPPFYLPPQQ<br>QPQPWQYPTQ<br>PPQLSPCQQF<br>GSCGVGSVGS<br>PFLGQCVEFL<br>RHQCSPAATP<br>YGSPQCQALQ<br>QQCCHQIRQV<br>EPLHRYQATY<br>GVVLQSFLQQ<br>QPQGELAALM<br>AAQVAQQLTA<br>MCGLQLQQPG<br>PCPCNAAAG<br>VY	KVLIVALAL<br>LALAASAASS<br>TSGGCGCQTP<br>PFHLP<br>QPWQYPTQ<br>PPQLSPCQQF<br>GSCGVGSVGS<br>PFLGQCVEFL<br>RHQCSPAATP<br>YGSPQCQALQ<br>QQCCHQI<br>HRYQATY<br>GVVLQSFLQQ<br>QPQGELAALM<br>AAQVAQQLTA<br>MCGLQLQQPG<br>PCPCNAAAG	VEFL<br>PLHRY<br>FLQQ<br>QPQGELAA<br>QVAQQL<br>GLQLQQ	KVLIVALAL<br>LALAASAASS<br>TSGGCGCQTP<br>PFHLPPPFY<br>PQQ<br>QPQPW<br>LSPCQQF<br>GSCGVGSVGS<br>PFL<br>CVEFL<br>CSPAATO<br>YGSPQCQAL<br>QCCHQIRQV<br>EPLHRYQATY<br>GVVLQSFLQQ<br>QPQGELAALM<br>AAQVAQQL<br>GLQLQQPG<br>PCPCNAAAG
Zein-19	Area%	11.3%	13.0%	25.5%	17.7%	1.2%	4.8%	13.6%	3.5%	31.3%	15.3%
	Coverage%	27.9%	44.2%	34.6%	67.5%	11.2%	67.9%	93.3%	87.1%	17.5%	44.6%
	Sequences	ATIFPQCSQA<br>PIAS<br>CEN<br>PALQPYRLQ<br>NQLSTLN<br>PAAYLQ<br>LLPFYQ<br>QFAANPATLL<br>QLQQL<br>LALT<br>FYQ	KIFSLLM<br>LLALSTC<br>IASLLPPYL<br>PSTTA<br>LS<br>PLLFQQ<br>SLVQSLVQTI<br>PLINQVA<br>QQQLL<br>PFSQL<br>QQQLLPFNQ<br>LAAL<br>QQILLPFSQ<br>QQQLLPFYQ<br>QFAA	KIFSLLM<br>LLALSTC<br>PIASLLPPYL<br>PSIIAS<br>AL<br>SLVQSLVQTI<br>RAQQL<br>QLVL<br>QQILLPF<br>LLPFYQ<br>QLQQLL<br>HIIGGAL<br>PL<br>QF<br>LL	MATKIFSLLM<br>LLALSTCVAN<br>ATIFP<br>PIASLLPPYL<br>PSIIASCICEN<br>PALQPYRLQQ<br>AIAASNIPLS<br>VQTI<br>RAQQLQ<br>QVALAN<br>LSPYSQQQ<br>LQQQLL<br>FPSQLATA<br>AYL<br>QQQILLPFS	IPLS<br>PLLF<br>LLPFYQ<br>QFA<br>PATLL<br>QLQQL	LLALSTCVAN<br>ATIFPQCSQA<br>PIASLLPPYL<br>PSIIASICEN<br>PALQPYRLQQ<br>AIAASNIPLS<br>PLLFQQS<br>RAQQLQQLVL<br>PLINQ<br>SPYSQQQQF<br>LPFNQLSTLN<br>PAAYLQQQLL<br>QQQQLLPENQ<br>LAALNPAAYL<br>ANRASFL	MATKIFSLLM<br>LLALSTCVAN<br>ATIFPQCSQA<br>PIASLLPPYL<br>PSIIASICEN<br>PALQPYRLQQ<br>AIAASNIPLS<br>PLLFQQSPAL<br>SLVQSLVQTI<br>RAQQLQQLVL<br>PLINQVALAN<br>LSP<br>QQQF<br>LPFNQLSTLN<br>PAAYLQQQLL	ATIFPQCSQA<br>PIASLLPPYL<br>PSIIASICEN<br>PALQPYRLQQ<br>AIAASNIPLS<br>PLLFQQSPAL<br>SLVQSLVQTI<br>RAQQLQQLVL<br>PLINQVALAN<br>LSPYSQQQQF<br>LPFNQLSTLN<br>PA<br>PFSQLATAYS<br>QQQQLLPENQ<br>PAAYL	MATKIFSLL<br>IAASNIPL<br>ALAN<br>LSP<br>QQQF<br>FNQ<br>LAA<br>LAAANRA	LLALSTCVAN<br>ATIFPQCSQA<br>PIASL<br>RLQQ<br>AIAA<br>NIPLS<br>PLL<br>VQSLVQTI<br>RA<br>PLINOVA<br>QQQF<br>QLLPENQ<br>QQQILL<br>AANRASFL<br>TQQ

			QLLPF<br>VL		NRASFL<br>TQQQLLPFYQ<br>QFAANP<br>QQLLPFVQ<br>LALTDPAASY<br>QQHIIG		TQQQLLP<br>ALLTDPAASY<br>QQHIIG	FPSQLATAYS<br>QQQQLLPENQ<br>LAALNPAAYL<br>QQQILLPFSQ<br>LAALNPAAYL<br>TQ<br>LPFYQ<br>QFAANPATLL<br>QLQQL<br>LALTDPAASY<br>QQ	QQQILLPFSQ<br>LAALNPAAYL<br>TQQQLLPFYQ<br>QFAANPATLL<br>QLQQLLPFVQ<br>LALTDPAASY<br>QQHIIGGALF		LLPFVQ<br>LALTD<br>LAA<br>LVL<br>YL
Glutenin	Area%<br>Coverage%<br>Sequences	23.4%<br>51.6%<br>MRVLLVA<br>HTSGGCGCQP<br>PPPVHL<br>HP<br>CPCQQPHPSP<br>CQLQGTCGVG<br>STPILGQCVE<br>FLRHQCSPTA<br>TPYSCPQCQC<br>LRQQCCQQLR<br>QQQPQSGQ<br>GLQQP<br>TPCPYAAAGG<br>IL	7.9%<br>89.2%<br>RVLLVALAL<br>LALAASATST<br>HTSG<br>CQP<br>PPPVHLPPPV<br>HLPPPVHLPP<br>PVHLPPPVHL<br>HVPPPVHLPP<br>PPCHYPT<br>RPQPHPQPHP<br>CPCQQPHPSP<br>STPILGOCVE<br>FLRHQCSPTA<br>LRQQCCQQLR<br>QVEPQHRYOA<br>IFGLVLQSIL<br>QQQPQSGQV<br>LAAQIAQQ<br>LTAMCGLQQP<br>TPC<br>TPYC	27.4%<br>34.1%<br>MRVLLVALAL<br>LALAA<br>TSGGCGCQ<br>HVPPPV<br>TQPP<br>RPQPHPQPHP<br>PILGQC<br>VLQSIL<br>QQQ<br>VA<br>GLLAAQIAQQ<br>LTAMC	34.3%<br>80.0%<br>MAAKIFSILM<br>LLALSACVLD<br>ATIFPQYSQA<br>PIAALL<br>PSMTASV<br>EN<br>PTLQPYRLQQ<br>NLPLS<br>PLLFQQSPAL<br>SLVQ<br>VQTI<br>RAQQLQQLVL<br>QVALAN<br>LSPYSQQQ<br>LQQQLL<br>PFSQLATA<br>FLPENQ<br>LAALNPAAYL<br>QQQILLPFGQ<br>LATTNRASFL<br>TQQQLLPFYQ<br>PATLL<br>QLQQLL<br>NPAAFY<br>QQHIIGGAI<br>QF	10.9%<br>47.5%<br>AASATST<br>HTSGGCGCQP<br>PP<br>VHLPPPV<br>HLPPPVHLPP<br>PVHLPPPVHL<br>PPPVHLPPPV<br>HVPPPVHL<br>PCHYPTQPP<br>CQQPHPSP<br>CSPTA<br>TPYCSP<br>QQQPQSGQ<br>RP<br>CQ<br>IL	15.1%<br>69.1%<br>RVLLVALAL<br>LALAASATST<br>HTSGGCGCQP<br>PPPVHLPPPV<br>CHYPTQPP<br>RPQPHPQPHP<br>CPCQQPHPSP<br>TCGVG<br>STPILGQCVE<br>FLRHQCSPTA<br>TPYCSPQCQS<br>LRQQCCQQLR<br>QVEPQHRYQA<br>IFGLVLQ<br>GLLAAQTAQQ<br>LTAMCGLQQP<br>TPCP	32.2%<br>96.9%<br>RVLLVALAL<br>LALAASATST<br>HTSGGCGCQP<br>PPPVHLPPPV<br>HLPPPVHLPP<br>PVHLPPPVHL<br>PPPVHLPPPV<br>HVPPPVHLPP<br>PPCHYPTQPP<br>RPQPHPQPHP<br>CPCQQPHPSP<br>CQLQGTCGVG<br>STPILGOCVE<br>FLRHQCSPTA<br>TPYCSPQCQS<br>LRQQCCQQLR<br>QVEPQHRYQA<br>IFGLVLQSIL<br>QQQPQSGQVA<br>GLLAAQIAQQ<br>LTAMCGLQQP<br>TPCPYAA	20.4%<br>76.7%<br>MAAKIFSILM<br>LLALSACVLD<br>HTSGGCGCQP<br>PPPVHLPPPV<br>HLPPPVHLPP<br>VPPPVHLPP<br>PPCHYPTQPP<br>RPQPHPQPHP<br>CPCQQPHPSP<br>CQLQGTCGVG<br>STPILGOCVE<br>TPYCSPQCQS<br>LRQQCCQQLR<br>HRYQA<br>GLVLQSI<br>QQQPQSGQVA<br>LTAMCGLQQP	10.5%<br>5.4%<br>VE<br>FLRH<br>PQSGQVA	31.7%<br>77.1%<br>VLLVALAL<br>LALAASATST<br>HTSGGCGCQP<br>PP<br>VHLPPPV<br>HLPPPVHLPP<br>PVHLPPPVHL<br>PPPVHLPPPV<br>HVPPPVHLPP<br>PPCHYPTQPP<br>RPQPHPQP<br>HPSP<br>CQLQGTCGVG<br>CVE<br>TPYCSPQCQS<br>LRQQCCQQL<br>PQSGQVA<br>GLLAAQIAQQ<br>LTAMCGLQQP<br>TPCPYAAA<br>ST<br>PLR
